# Fetal Urine Production in Late Pregnancy

**DOI:** 10.5402/2011/345431

**Published:** 2011-06-30

**Authors:** Robert H. Stigter, Eduard J. H. Mulder, Hein W. Bruinse, Gerard H. A. Visser

**Affiliations:** Department of Perinatology & Gynecology, Wilhelmina Children's Hospital, University Medical Centre Utrecht, Lundlaan 6, 3584 EA Utrecht, The Netherlands

## Abstract

*Objective*. Hourly fetal urine production rate (HFUPR) was studied in relation to both gestational age and the onset of spontaneous labor in normal term human pregnancies. *Methods*. Serial volume measurements were obtained from longitudinal ultrasound images of the fetal bladder at 1–5-minute intervals, and HFUPR was subsequently calculated. A total of 178 adequate bladder-filling cycles were recorded in 112 women, and the amniotic fluid index (AFI) was assessed. *Results*. HFUPR did not change significantly between 37 and 42 weeks' gestation. However, HFUPR decreased during the last 14 days prior to the onset of spontaneous labor (*P* < 0.005). No significant correlation was found between HFUPR and AFI, neither when measured at the same time nor when HFUPR and AFI were measured at various intervals in time. *Conclusion*. HFUPR falls before and in relation to the time of onset of labor rather than in relation to gestational age.

## 1. Introduction

Prolongation of pregnancy beyond term is often associated with oligohydramnios and with increased perinatal mortality and morbidity [1–3]. Several studies have shown a relationship between the presence of oligohydramnios and an unfavourable pregnancy outcome, regardless of gestational age [1, 4–7]. The ultrasonographic assessment of the quantity of amniotic fluid, often expressed as the amniotic fluid index (AFI), is an important parameter of fetal monitoring in prolonged pregnancy [7]. The AFI, the sum of the deepest amniotic fluid pockets in four abdominal quadrants, has been shown to be a reproducible test and was found to correlate well with volumes determined by dilution methods [8, 9]. A reduction in the amount of amniotic fluid in pregnancies beyond term is often reported. This has been shown in invasive studies of cross-sectional design using a variety of dilution techniques [10] and by ultrasound measurement of the AFI in longitudinal studies [11, 12]. Recently we found no reduction in the AFI with advancing gestation in accurately dated pregnancies, but a significant reduction in relation to the time of onset of spontaneous labor [13]. 

The quantity of amniotic fluid in third trimester pregnancy is the result of contributions from fetal urine and the secretion of fetal lung liquid on the one hand, and the removal of fluid by fetal swallowing and resorption through the fetal membranes on the other hand [14]. The mechanisms responsible for the regulation of the amniotic fluid volume are unknown. It is often assumed that the features of postterm pregnancy are the result of placental insufficiency and growth delay [15–18]. There are similarities between clinical features of intrauterine growth retardation (IUGR) occurring earlier in pregnancy and postterm pregnancy. In the growth-retarded fetus changes in the circulation occur, favouring the brain, adrenals, and coronary arteries at the expense of other organ systems such as the digestive system and renal tract, a phenomenon known as “brain sparing” or “redistribution.” In the growth-retarded fetus the reduced perfusion of the fetal kidneys is deemed responsible for a reduction of fetal urine output and a subsequent reduction of the amniotic fluid volume. 

It was the purpose of this study to examine hourly fetal urine production rate (HFUPR) in relation to both gestational age and the time of onset of spontaneous labor in term pregnancies. We also examined the relationship between HFUPR and AFI.

## 2. Patients and Methods

### 2.1. Patients

The study was approved by the ethics committee of University Medical Centre Utrecht, and informed consent was obtained from all women. A total of 142 women with uncomplicated singleton pregnancies were recruited from the outpatient clinic. Hourly fetal urine production rate (HFUPR) and amniotic fluid index (AFI) were assessed at weekly intervals between 37 and 41 weeks and then twice weekly until delivery. AFI was determined during 220 sessions, and HFUPR was assessed in 200 recordings. HFUPR recordings did not fulfill predefined quality criteria (see below) in 13 sessions, leaving 187 HFUPR measurements in 117 women for further analysis. A single HFUPR measurement was available from 75 of the 117 women, while more than one HFUPR recording was available from 42 women (23 had 2, 15 had 3, one had 4, one had 5, and 2 had 6 recordings each).

The women had been recruited at a mean gestational age of 277 days (range 249–295 days). Sixty-nine (59%) women were nulliparous. Gestational age (GA) was calculated from a first-trimester crown-rump length (CRL) measurement in 95 (81%) cases or from the last menstrual period (LMP) if no first trimester ultrasound measurements were available. Gestational age was based on uncertain LMP in 4 cases. Labor onset was spontaneous in 89 (76%) women. Mean number of days prior to spontaneous delivery was 10 (SD 7, range 0–35). Gestational age at delivery ranged between 266 and 302 days (mean 287 days). Mean birth weight was 3552 (SD 471) grams, and 48 girls and 69 boys were born. The 5-minute Apgar score was ≥7 in all cases.

### 2.2. Methods

All examinations were performed by a single investigator (RHS) by means of a colour Doppler ultrasound machine (Toshiba SSH 140A, Toshiba Medical Systems Division, Tokyo, Japan) fitted with a 3.75 MHz curved array transducer. Examinations were all carried out between 14:00 and 17:00 h, and care was taken to perform repeat measurements in each individual at the same time of day [19]. The AFI was determined at the beginning of each session as described by Phelan et al. [20], with the woman in supine position. On completion of the AFI measurement the woman was placed in a more comfortable semi recumbent position. Ultrasound biometry was next performed to estimate fetal weight. A CTG monitor (Hewlett Packard 8040A) was used for continuous recording of the fetal heart rate throughout the remainder of the investigation (mean duration 46 min, range 21–83 min). All fetal heart rate recordings showed normal patterns. 

Fetal bladder volume measurements were performed using the method introduced by Hedriana and Moore which showed a better correlation with known bladder volumes than previously used methods [21]. We adapted the technique by averaging the results of bladder volume calculations from the equations for exact coronal and sagittal planes as described previously [22]. A longitudinal section was obtained of the entire fetus and the largest outline of the fetal bladder selected by making parallel scans on either side of the original plane, as described by Campbell et al. [23]. Images were obtained at 1–5-minute intervals, printed on a strip chart recorder, and subsequently scanned for off-line analysis. The surface area of the longitudinal section of the fetal bladder was calculated by tracing the outline using a computer software program (NIH image) on a standard personal computer. Fetal bladder volumes were calculated from the equation Vol. = 0.84 + 1.23 × bladder area, the mathematical average of the equations for exact sagittal and coronal measurements provided by Hedriana and Moore [21] and Stigter et al. [22]. HFUPR was calculated from the slope of the regression line through the individual bladder volume measurements as described by Rabinowitz et al. [24]. Cases were included only if a minimum of 6 individual bladder volume measurements (mean number 13, range 6–43) were available, and the interval between the first and last measurements of a series of bladder volume measurements was ≥15 min (mean interval 29 min, range 15–59 min).

### 2.3. Data Analysis

The relationship between HFUPR and gestational age was examined by using only the last HFUPR measurement of any individual where gestational age had been calculated from a first trimester CRL measurement. When examining the relationship between HFUPR and the time of onset of labor, the accuracy of gestational age was not essential, and again only the last measurement from each individual who labored spontaneously was included.

The relationship between HFUPR and AFI at various time points in the cases with repeat measurements was analysed by randomly selecting combinations for each individual out of 108 possible combinations.

SPSS for Windows (version 16.01, SPSS Inc., Chicago, Ill) was used for data management and statistical analysis. Data were analysed by linear regression analysis, 1-way ANOVA, or linear mixed model analysis (in case of repeated measurements). Significance was assumed at *P* < 0.05.

## 3. Results

**Figure 1:**
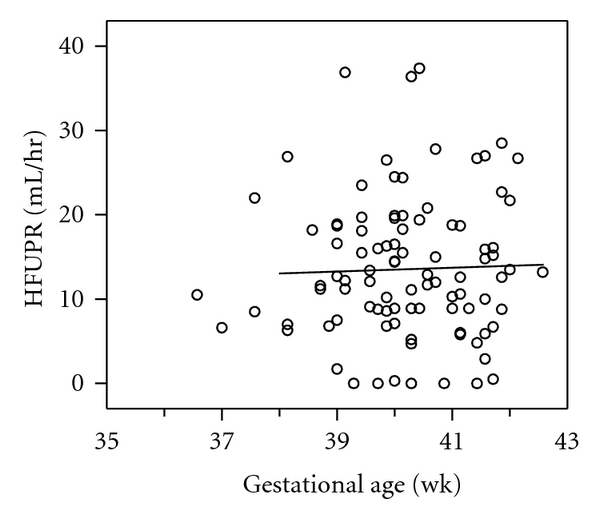
Relationship between hourly fetal urine production rate (HFUPR) and gestational age in 95 cases with accurately dated pregnancy. Only the last measurement from an individual case was used if more than one measurement was available.

The last HFUPR measurements obtained from 95 individuals with accurately dated pregnancies are shown in Figure 1. There was no significant change in HFUPR between 38 and 42 weeks' gestation. The same was true for HFUPR corrected for estimated fetal weight (data not shown). Linear mixed model analysis for the group of 42 women with at least 2 repeated measurements also showed no significant change in HFUPR with advancing gestation (*β* = −0.10; SE 0.13; df = 80; *P* = 0.44).

**Figure 2:**
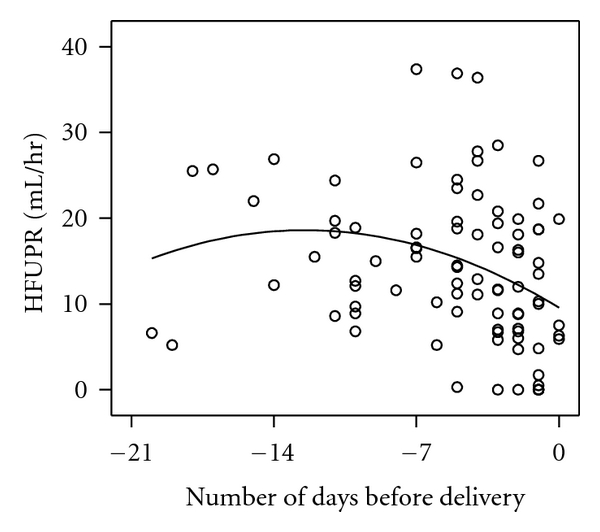
Relationship between hourly fetal urine production rate (HFUPR) and time of onset of spontaneous labor (*n* = 89 cases). Only the last measurement from an individual case was used if more than one measurement was available.

The last HFUPR measurements recorded in each individual who labored spontaneously (*n* = 89) are shown in Figure 2. A polynomial regression line fitted the data best: *y* = 9.56 − 1.45× (number of days to delivery) −0.058 × (number of days to delivery)^2^. HFUPR decreased linearly over the last 14 days before the onset of labor (*β* = −0.62; *n* = 80, *P* < 0.005), especially during the last 3 days (Table 1). Linear mixed model analysis in the group of women with ≥2 repeated measurements also demonstrated a significant fall in HFUPR during the last two weeks before delivery (*β* = −0.70; SE 0.31; df = 44; *P* < 0.05). Similar results were obtained for HFUPR corrected for estimated fetal weight (data not shown).

**Figure 3:**
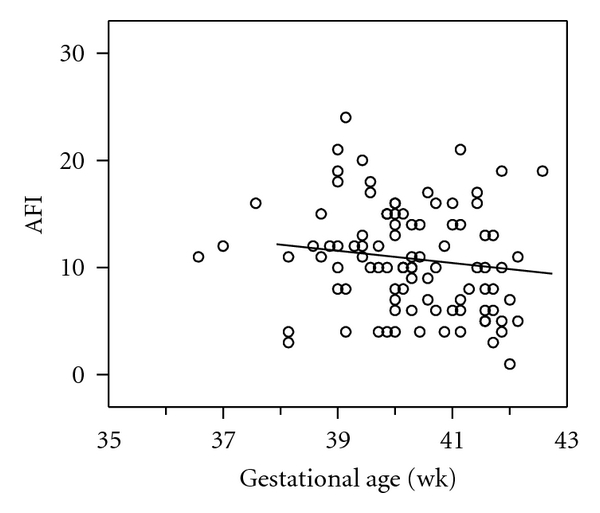
Relationship between amniotic fluid index (AFI) and gestational age in 95 cases with accurately dated pregnancy. Only the last measurement from an individual case was used if more than one measurement was available.

**Figure 4:**
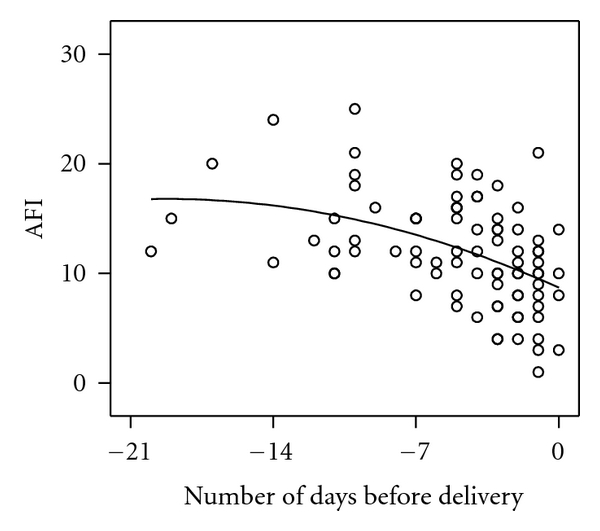
Relationship between amniotic fluid index (AFI) and time of onset of spontaneous labor (*n* = 87 cases). Only the last measurement from an individual case was used if more than one measurement was available.

AFI values were normalised for gestational age based on the normal values described by Nwosu et al. [12] and expressed as *z*-score. The AFI values and their *z*-scores were highly correlated (*R* = 0.97; *P* < 0.0001; *n* = 197). In women with accurately dated pregnancies, AFI values obtained during the final session did not change significantly between 38 and 42 weeks' gestation (Figure 3; *β* = −0.24, *P* = 0.094, *n* = 92). A similar observation was made in the cases with ≥2 repeated measurements (*β* = −0.09, SE 0.06, df = 57, *P* = 0.12). However, in the cases with spontaneous labor, the last AFI values declined over the 14 days prior to delivery (Figure 4; *β* = −0.62, *P* < 0.001, *n* = 82), which was also true for individual cases with repeated measurements over the last 14 days (*β* = −0.13, SE 0.06, df = 42, *P* < 0.05).

We examined the relationship between HFUPR and AFI (absolute values and *z*-scores) during the same session in all cases and the effect of various time intervals between sessions in the serial data sets from 42 cases (Table 2). HFUPR values were not corrected for gestational age since changes over the time intervals studied are negligible. No significant correlation was found between the HFUPR and AFI values obtained during the same session (*R* = 0.08 and *R* = 0.07 for *z*-scores; *n* = 183). Good correlations were found between serial AFI measurements within individuals at intervals of up to two weeks, but not for HFUPR values obtained within individuals at various time intervals (Table 2). HFUPR and AFI (or *z*-scores) were not significantly correlated for any of the studied time intervals (Table 2).

## 4. Discussion

Using an adaptation of the method introduced by Hedriana and Moore [21], we found no significant change of the hourly fetal urine production rate with advancing gestational age between 38 and 42 weeks' gestation in accurately dated pregnancies, with or without correction for estimated fetal weight. However, we did find a significant reduction of the HFUPR during the last 14 days prior to the onset of spontaneous labor. No significant correlation was found between fetal urine production rate and AFI.

Previous reports on fetal urine production rates, using the measurement technique introduced by Campbell et al. [23], are flawed because bladder volume measurements were made too infrequently. Complete or partial emptying of the fetal bladder must have been overlooked in between consecutive measurements, leading to an underestimation of fetal urine output. The subsequent modifications introduced by Rabinowitz et al. [24] should have overcome this problem, yet were found by Hedriana and Moore [21] to result in a considerable overestimation of fetal urine production. In an earlier study, using the method introduced by Rabinowitz et al. [24], we found a significant positive correlation between calculated HFUPR and the maximum bladder volume at the end of a series of measurements, suggesting a progressive error [25]. Hedriana and Moore [21] showed a good correlation between known bladder volumes and volumes calculated from a sagittal or coronal area measurement only (*R* = 0.95 and *R* = 0.94, resp.). The accuracy of their technique to determine HFUPR showed no improvement beyond 6 individual measurements of bladder volume in a filling cycle. Rabinowitz et al. [24] did not define a minimum number of individual volume measurements to reliably calculate HFUPR, nor did they suggest a minimum duration for the period of observation, which may explain the unrealistic values of more than 100 mL/h, observed in some studies. The maximum bladder volumes and urine production rates found in our study are more in keeping with neonatal data and with observed urine production rates in animal experiments. We found no indication for a reduction in fetal urine production with increasing gestation, as reported in a number of studies [26, 27]. No previous study has examined fetal urine production in relation to the time of onset of labor. However, Wlodek et al. found no change in the number of voids between 125 and 144 days' gestation in sheep fetuses but a significant decrease in the number of voids during the last 5 days before onset of labor, which suggests a possible decrease in the rate of urine production during this period [28].

In contrast with others, we found no significant relationship between HFUPR and AFI measured at the same time [29]. It is reasonable to assume that a decrease in fetal urine production does not have an immediate effect on the amount of amniotic fluid as a number of other mechanisms are involved in maintaining the quantity of amniotic fluid at a constant level. An example of the potential of these compensatory mechanisms can be found in a study by Minei and Suzuki [30], where occlusion of the oesophagus in primate fetuses resulted in only a transient increase of the amniotic fluid volume. In our study AFI measurements remained constant for periods of up to two weeks, but HFUPR measurements showed considerable variation within individuals. The effect of fetal behavioural state changes on the variation in HFUPR measurements in near-term pregnancy, as suggested in one study [31], was found to be the result of the progressive measurement error with increasing volumes inherent to the Rabinowitz' method and the fact that the fetus usually voids at the transition from state 1F to 2F [25]. The variability in HFUPR values within individuals observed in our study could still be the result of measurement errors, may reflect true short-term fluctuations of fetal urine production or a combination of both, and this precludes the useful analysis of changes within individuals. 

Recently, we found a significant reduction in AFI in relation to the onset of labor, but not in relation to gestational age in accurately dated pregnancies [13]. We conclude that it is likely that the reduction in fetal urine production contributes to the reduction in amniotic fluid in the period before onset of labor, but the large variation in HFUPR measurements prevents the establishment of a direct relationship within individuals. The reduction in fetal urine production and amniotic fluid is related to physiological changes occurring in preparation of labor and is not related to gestational age. The reduction in fetal urine production may at least in part be the result of circulatory “redistribution” and subsequent reduced renal perfusion that was observed in these pregnancies and reported previously [32].

## Figures and Tables

**Table 1:** Final HFUPR measurement and time to onset of spontaneous labor with (mL/kg/h) and without correction for estimated fetal weight (mL/h). Data are presented as 3-day categories. No data were available on estimated fetal weight in 5 cases.

Number of days to onset delivery	HFUPR (mL/h)		HFUPR (mL/kg/h)	
	No.	mean (SEM)	No.	mean (SEM)
−9 to −11	10	14.1 (1.7)	10	4.7 (0.5)
−6 to −8	10	17.5 (3.2)	8	5.9 (1.3)
−3 to −5	29	16.5 (1.7)	29	5.3 (0.6)
0 to −2	30	10.2 (1.4)*	27	3.5 (0.5)**

*P < 0.02; **P < 0.05 compared with the other 3-day categories (1-way ANOVA with Bonferroni correction).

**Table 2:** Relationship between measurements of AFI and HFUPR in individuals with repeat measurements at various time intervals. Data are presented as correlation coefficient (*R*) and *P* values, *n* = number of measurements.

Median interval (days)	Range (days)	*n*	AFI at time 1 versus AFI at time 2		HFUPR at time 1 versus HFUPR at time 2		HFUPR at time 1 versus AFI at time 2	
			*R*	*P*	*R*	*P*	*R*	*P*
3	1–4	16	0.84	<0.0001	0.21	NS	−0.34	NS
7	5–7	24	0.55	<0.005	−0.27	NS	0.03	NS
10	8–13	20	0.53	<0.02	−0.07	NS	0.25	NS
17	14–21	16	0.51	<0.05	−0.17	NS	−0.29	NS
